# Gender Bias and Diagnostic Delays in Young Women: A Narrative Review

**DOI:** 10.7759/cureus.100004

**Published:** 2025-12-24

**Authors:** Karishma Harrilal-Maharaj

**Affiliations:** 1 Internal Medicine, Basildon University Hospital, Basildon, GBR

**Keywords:** diagnostic delay, diagnostic errors, gender bias, healthcare disparities, young women

## Abstract

Gender bias continues to influence diagnostic pathways for young women, contributing to delayed recognition of serious neurological, cardiovascular, autoimmune, and pain-related conditions. Symptoms such as visual disturbances, chest discomfort, fatigue, and multisystem complaints are frequently misattributed to stress, psychosocial factors, or benign causes. This narrative review synthesises existing literature on diagnostic disparities affecting young women across multiple specialities. Evidence highlights longer diagnostic intervals for conditions such as multiple sclerosis, optic neuritis, acute coronary syndromes, lupus, and chronic pain syndromes. Contributing factors include the under-recognition of sex-specific symptom profiles, historical male-centred research models, and cognitive heuristics that disproportionately categorise women’s symptoms as functional or anxiety-related. International comparisons reveal narrower diagnostic gaps in Scandinavian systems with structured equity frameworks, whereas data from Australia and other regions demonstrate persistent triage and investigation differences that disadvantage young women. The consequences of delayed diagnosis include irreversible organ damage, increased long-term disease burden, psychological distress, and reduced quality of life. This review emphasises the need for bias-aware clinical assessment, improved education on sex-based differences, and system-level strategies to ensure that young women’s symptoms are recognised and investigated promptly. Addressing diagnostic gender bias is essential for improving patient outcomes and achieving equity within modern healthcare.

## Introduction and background

Gender-based disparities in diagnosis represent a longstanding challenge across global healthcare systems, shaping how symptoms in women are interpreted, investigated, and prioritised [1,2]. Diagnostic gender bias refers to systematic differences in clinician perception or decision-making that contribute to delayed, missed, or inaccurate diagnoses in women, independent of disease severity or presentation [3]. Large-scale studies consistently demonstrate that women experience longer diagnostic intervals for multiple acute and chronic conditions, including cardiovascular disease, autoimmune disorders, neurological conditions, and chronic pain syndromes [4-6]. These disparities often appear early in life; young women are disproportionately affected by symptom minimisation and misattribution, particularly when presenting with visual disturbances, fatigue, multisystem symptoms, or chest discomfort [7,8]. For the purposes of this review, “young women” broadly refers to women in early adulthood and reproductive age, recognising that age cutoffs vary across studies and clinical contexts.

Epidemiological data highlight the scale of the problem. Women with acute coronary syndromes are more likely to be discharged from emergency departments while actively experiencing myocardial infarction and have longer times to reperfusion compared to men, especially in younger age groups [9-11]. In neurology, women with early symptoms of multiple sclerosis, optic neuritis, or migraine frequently report prolonged pathways before receiving neuroimaging or specialist assessment, with symptoms such as paresthesia, visual blurring, or imbalance often reframed as stress or anxiety [12-14]. Autoimmune diseases, including systemic lupus erythematosus, rheumatoid arthritis, and thyroid autoimmune disorders, each disproportionately affecting women, are associated with diagnostic delays linked to misinterpreted early symptoms and inconsistent referral pathways [15-17]. Such delays contribute to irreversible organ damage, preventable disability, and poorer long-term outcomes [18].

A growing body of research points to the role of structural and cognitive mechanisms underpinning these disparities. Historically male-centric research frameworks, limited inclusion of women in foundational clinical trials, and reliance on symptom templates derived from male populations all contribute to under-recognition of sex-specific presentations [19-21]. Cognitive heuristics, such as the premature attribution of unexplained symptoms in young women to functional disorders, anxiety, or psychosomatic causes, further compound diagnostic inequity and delay escalation of care [22,23]. The underestimation of women's pain, reduced thresholds for psychologisation, and variations in triage categorisation in emergency settings have also been repeatedly documented across international studies [24-26].

Intersectional factors contribute additional layers of complexity. Women of colour face compounded diagnostic barriers due to overlapping effects of race, gender, and socioeconomic disadvantage, resulting in higher rates of missed or delayed diagnoses across cardiovascular, neurological, and autoimmune conditions [27,28]. Younger women in particular experience a heightened risk of diagnostic overshadowing, with studies showing that their symptoms are more likely to be dismissed or attributed to lifestyle factors [29]. International comparisons further illustrate the impact of healthcare infrastructure: countries with strong gender-equity frameworks and standardised triage protocols, such as Sweden and Norway, demonstrate narrower diagnostic gaps, whereas Australian and US data reveal persistent disparities in emergency assessment, cardiovascular investigation, and autoimmune disease recognition [30-33].

Despite the expanding literature on gender disparities in healthcare, diagnostic inequity in young women remains under-synthesised across specialities, with few reviews integrating evidence spanning neurology, cardiology, autoimmune medicine, emergency medicine, and pain medicine. These delays result in irreversible disease progression, psychological distress, increased healthcare utilisation, and long-term socioeconomic consequences [34-36].

Accordingly, this narrative review aims to synthesise the current literature examining diagnostic gender bias in young women across multiple medical specialities. It highlights consistent patterns of symptom misattribution, identifies system-level contributors to diagnostic delay, and outlines strategies to promote equitable, bias-aware clinical assessment [37-40].

## Review

Methods

This narrative review was developed using a targeted, non-systematic search of the literature. Electronic databases, including PubMed, Embase, and Google Scholar, were searched using combinations of terms such as “diagnostic delay”, “gender bias”, “young women”, “neurology”, “cardiovascular disease”, “autoimmune disease”, “emergency medicine”, “pain”, and “health disparities”. The literature search focused on publications from January 2000 to December 2024. Earlier seminal and theoretical works were included where necessary to provide conceptual context, particularly in relation to gender bias and intersectionality. Additional articles were identified through citation chaining of key reviews and landmark studies. Priority was given to observational cohort studies, registry data, meta-analyses, and qualitative work exploring clinician and patient perspectives on diagnostic pathways. Given the heterogeneity of study designs, populations, and outcome measures, findings were synthesised thematically rather than subjected to formal meta-analysis [2,3]. Consistent with a narrative review approach, no formal risk-of-bias assessment was undertaken. The subsequent sections are organised thematically by clinical speciality to reflect common diagnostic pathways and mechanisms of bias.

Neurological presentations in young women

Neurological disorders provide some of the clearest examples of diagnostic gender bias in young women. Conditions such as multiple sclerosis, optic neuritis, migraine, functional neurological disorder, and autoimmune demyelinating diseases frequently present with non-specific or fluctuating symptoms, including fatigue, sensory disturbance, visual changes, and imbalance [12-14]. In young women, these early manifestations are often interpreted through a psychosocial lens, attributed to stress, mood disturbance, or musculoskeletal strain rather than prompting timely neuroimaging or specialist referral [7,12].

Several observational studies have reported longer times to diagnosis and treatment initiation for women with demyelinating disease compared with men, even when symptom severity is similar [12-16]. Early visual symptoms, including unilateral blurring, pain on eye movement, or colour desaturation, may be framed as migraine aura, refractive error, or functional complaint, particularly when neurological examination is subtle or when women are perceived as “too young” for serious neurological disease [13,14]. Similar patterns are seen in headache disorders: women are more likely to have recurrent attendances for migraine or “stress headache” before secondary causes are excluded [4,12,22].

These delays are clinically significant. In inflammatory demyelinating conditions, early recognition and treatment are associated with improved visual and neurological outcomes, while prolonged inflammatory activity increases the risk of permanent disability [14-16,18]. Failure to investigate persistent or evolving neurological symptoms in young women, therefore, represents a critical point at which gendered assumptions can translate directly into preventable morbidity.

Cardiovascular disease and atypical presentations

Cardiovascular disease has traditionally been conceptualised as a male condition, with classic teaching centred on middle-aged men presenting with exertional chest pain. This historical framing has contributed to systematic under-recognition of ischaemic heart disease in women, particularly younger women, whose symptoms more commonly include dyspnoea, fatigue, back pain, nausea, and atypical chest discomfort [4,9,10]. Large registry and emergency department datasets consistently show that women are less likely than men to receive timely electrocardiography, biomarker testing, or cardiology consultation when presenting with possible acute coronary syndromes [9-11].

Young women are at particular risk. Studies have demonstrated higher rates of discharge from emergency departments without a cardiac diagnosis among women later shown to have myocardial infarction or unstable angina, even after adjustment for risk factors and presentation characteristics [9-11,24]. Explanations offered by clinicians frequently invoke anxiety, panic disorder, or musculoskeletal pain, reflecting both the non-classic symptom profile and implicit sex stereotypes regarding cardiovascular risk [19-21]. These patterns contribute to longer door-to-needle times, reduced use of guideline-directed therapies, and worse short- and long-term outcomes in women [9-11,24].

Microvascular angina and spontaneous coronary artery dissection, conditions more prevalent in women, may also be under-recognised, particularly when angiography is normal or equivocal [10,19]. Without a structured, sex-sensitive approach to chest pain assessment, young women remain vulnerable to diagnostic closure once life-threatening causes are deemed “unlikely” on the basis of age, sex, or emotional state rather than objective risk.

Autoimmune and rheumatologic disease

Autoimmune diseases disproportionately affect women, especially during the reproductive years, yet diagnostic pathways are often slow and fragmented [4,15-17]. Conditions such as systemic lupus erythematosus, rheumatoid arthritis, autoimmune thyroid disease, and connective tissue disorders commonly begin with non-specific features, such as fatigue, arthralgia, rash, and low-grade fever that overlap with benign or functional diagnoses. In young women, these symptoms may be attributed to stress, lifestyle, or mood, delaying appropriate serological testing and referral to rheumatology [15-17].

Literature from multiple health systems describes diagnostic intervals for lupus and other systemic autoimmune conditions spanning several years from symptom onset, with women frequently reporting repeated consultations before their concerns were taken seriously or investigated comprehensively [15-18,34]. Over this period, ongoing inflammation contributes to irreversible organ damage, including renal impairment, cardiovascular disease, and neurological involvement. For thyroid disease, weight change, menstrual irregularity, mood disturbance, and fatigue are often framed as primary psychiatric or situational problems before endocrine causes are considered, despite clear sex differences in prevalence [15,16].

These patterns illustrate how historically male-centric research frameworks and diagnostic criteria, developed using predominantly male cohorts, can fail to capture the complexity of autoimmune presentations in young women [19-21]. Without active attention to sex-specific symptom constellations and clustering, diagnostic bias persists even in ostensibly protocol-driven specialities.

Pain, functional labels, and psychologisation

Gender differences in the assessment and treatment of pain are well documented. Experimental and clinical studies suggest that women’s reports of pain are more likely to be downplayed, considered exaggerated, or explained by emotional factors compared with men presenting with similar complaints [24-26]. This bias extends to both acute and chronic pain syndromes, with women more frequently receiving sedatives or anxiolytics rather than thorough diagnostic work-ups or adequate analgesia [24,25].

For young women, the threshold for assigning “functional”, “somatic symptom”, or “medically unexplained” labels appears lower than for male patients [22,23]. Chronic pelvic pain, abdominal pain, and musculoskeletal complaints may be attributed to stress, irritable bowel syndrome, or primary dysmenorrhoea without systematic evaluation for endometriosis, inflammatory bowel disease, or other organic causes [24-26]. While functional diagnoses are sometimes appropriate and can guide legitimate management, premature closure on this explanation can curtail further investigation and shape subsequent clinician-patient encounters, with future symptoms seen through the lens of an established psychosomatic narrative.

The impact of such psychologisation goes beyond missed diagnoses. Qualitative work indicates that women who feel their pain has been dismissed or minimised often lose trust in healthcare providers, delay re-presentation, or disengage from services altogether, increasing the risk that serious underlying pathology will be detected only at an advanced stage [23-26,36].

Emergency medicine, triage, and diagnostic pathways

Emergency departments represent critical decision points where gender bias can manifest quickly and with high stakes. Studies examining triage scores and process measures have found that women, including young women, are sometimes assigned lower acuity categories than men with similar vital signs and presenting complaints, resulting in longer waiting times and reduced access to urgent investigations [24-26,30]. In the context of chest pain, neurological symptoms, or severe pain, these discrepancies contribute directly to diagnostic delay [9-11,24].

The combination of crowding, time pressure, and limited continuity of information amplifies reliance on heuristics and first impressions. Young women presenting repeatedly with non-specific or fluctuating symptoms may be informally labelled as “frequent attenders” or “anxious”, influencing subsequent clinicians’ willingness to pursue new differentials or escalate care [22,23]. Documentation that emphasises psychiatric or behavioural attributes can further bias later assessments, entrenching a cycle of under-investigation.

Conversely, structured triage tools and protocolised pathways have been shown to reduce subjective variation and narrow gender gaps in some settings, particularly when sex-specific thresholds and symptom patterns are incorporated [30-33]. However, implementation is inconsistent, and even standardised tools can be overridden by informal clinical judgement.

Intersectional and age-related disparities

Gender does not operate in isolation. Intersectional analyses reveal that women from ethnic minority backgrounds, lower socioeconomic strata, and marginalised communities experience compounded barriers to timely diagnosis [27-29]. Language discordance, mistrust of healthcare institutions, and differential treatment based on race or class all intersect with gender to shape diagnostic trajectories. For example, women of colour may be less likely to receive advanced imaging, specialist referral, or guideline-directed investigations even when controlling for insurance status and comorbidities [27,28].

Age further modifies risk. Young women are often perceived as “low risk” for many serious conditions, including stroke, myocardial infarction, and malignancy, leading clinicians to anchor on benign or psychological explanations [7-9,29]. When these women also occupy minority or disadvantaged social positions, the likelihood of diagnostic overshadowing increases. Qualitative studies describe women reporting that they were “not believed”, “reassured without tests”, or told they were “too young for something serious”, only to be diagnosed later with advanced disease [23,27-29,36].

Recognising these intersectional dynamics is essential for designing equitable interventions; generic gender-sensitive strategies may fail to reach those at greatest risk if they do not explicitly address race, class, and other structural determinants of health.

International comparisons of diagnostic pathways

Cross-national comparisons suggest that system design and policy can moderate the impact of gender bias. Scandinavian countries, which have invested in gender equity policies and standardised clinical pathways, report smaller sex differences in some diagnostic intervals, particularly for cardiovascular and neurological conditions [30-33]. Uniform access to primary care, robust electronic health records, and structured referral criteria appear to reduce reliance on subjective impressions and personal networks.

In contrast, data from more fragmented health systems, including parts of Australia, the United Kingdom, and North America, demonstrate persistent gender gaps in emergency triage, investigation rates, and specialist access [30-33]. Out-of-hours arrangements, copayment structures, and variable primary care continuity can exacerbate these disparities, particularly for young women juggling precarious employment or caregiving responsibilities.

These international patterns highlight that diagnostic gender bias is not an immutable feature of clinical practice but is shaped by organisational, financial, and policy environments. Systems that support continuity, standardisation, and equity tend to show narrower gender gaps, providing a model for reform elsewhere.

Consequences of diagnostic delay in young women

The clinical consequences of diagnostic delay are wide-ranging. In neurology, delayed recognition of demyelinating disease, optic neuritis, or autoimmune encephalitis can result in irreversible visual loss, progressive disability, and reduced response to disease-modifying therapies [12-18]. In cardiology, postponing or missing the diagnosis of acute coronary syndromes increases infarct size, heart failure risk, and mortality [9-11,24]. For autoimmune and rheumatologic conditions, prolonged uncontrolled inflammation accelerates organ damage, cardiovascular comorbidity, and functional impairment [15-18].

Beyond organ-specific outcomes, repeated experiences of dismissal or misattribution can precipitate psychological distress, demoralisation, and mistrust in healthcare providers [23-26,34-36]. Women may delay seeking care for new or worsening symptoms, attempt to self-manage serious conditions, or disengage from follow-up, further compounding risk. Economic and social consequences, including lost employment, caregiving disruption, and financial strain, disproportionately affect young women at pivotal life stages.

From a health system perspective, diagnostic delay leads to increased emergency use, more complex hospital admissions, and higher long-term costs as conditions present later and with greater severity [4,18,34,35]. Addressing gender bias is, therefore, not solely an ethical imperative but also a pragmatic strategy to improve efficiency and sustainability.

To synthesise the cross-speciality findings described above, Table 1 summarises the major clinical presentations in young women, the mechanisms of diagnostic delay across different specialities, and the resulting clinical consequences.

**Table 1:** Clinical presentations in young women and mechanisms of diagnostic delay. Synthesised from multiple observational, qualitative, and registry-based studies [4,7,9-18,22-26]. MSK: musculoskeletal; IBD: inflammatory bowel disease; CVD: cardiovascular disease; CNS: central nervous system.

Domain/speciality	Typical presentations in young women	Common misattributions/causes of delay	Consequences of delay
Neurology	Visual blurring, sensory loss, fatigue, imbalance	Attributed to stress, migraine, and anxiety; underuse of MRI	Missed steroid window; irreversible neurological deficit
Cardiology	Dyspnoea, nausea, atypical chest pain, back pain	Anchoring on anxiety/panic; “too young” bias; less ECG/troponin	Larger infarcts, delayed reperfusion, and higher mortality
Autoimmune/rheumatology	Fatigue, arthralgia, rash, low-grade fever	Seen as lifestyle/mood-related; delayed serology and referral	Organ damage (renal/CNS), disability, CVD risk
Pain/gynaecology	Pelvic pain, abdominal pain, chronic MSK pain	Rapid assignment of functional labels; inadequate assessment	Missed endometriosis, IBD, malignancy, chronic pain
Emergency medicine	Recurrent vague symptoms, headache, chest pain	Lower triage acuity; dismissal; documentation bias	Repeated attendances; preventable deterioration

Building on this clinical summary, Table 2 outlines the key system-level contributors to diagnostic gender bias, including triage processes, care fragmentation, psychologisation, and the influence of male-centred diagnostic pathways.

**Table 2:** System-level contributors to diagnostic gender bias in young women. Derived from comparative policy analyses, ED datasets, and gender-disparity research [1,4,19-21,27-33]. ED: emergency department; SES: socioeconomic status; ACS: acute coronary syndrome.

System factor	How it contributes to the delay	Illustrative mechanisms
Triage processes	Women are assigned a lower urgency despite comparable symptoms	Heuristics; “low-risk” assumptions based on age/sex
Fragmented care	Loss of continuity; symptoms interpreted in isolation	Inconsistent follow-up; incomplete history transfer
Implicit bias & psychologisation	Symptoms reframed as stress/anxiety	Earlier attribution to psychosomatic causes
Male-centred diagnostic pathways	Criteria developed using male cohorts; atypical female patterns overlooked	ACS criteria; autoimmune scoring systems
Sociocultural/intersectional factors	Compounded disadvantage for minority/low-SES women	Language barriers, racial bias, and reduced access to imaging/specialists

Strategies to reduce diagnostic gender bias

The literature suggests multiple, complementary strategies to mitigate diagnostic gender bias in young women. At the clinician level, education on sex- and gender-specific symptom profiles, explicit training in cognitive debiasing, and reflection on implicit attitudes may help reduce reliance on stereotypes [19-23]. Tools such as checklists, decision aids, and structured differential diagnosis prompts can help ensure that serious conditions remain under consideration even when psychosocial factors are present.

At the system level, implementing standardised triage protocols, sex-sensitive clinical pathways, and audit-and-feedback mechanisms can highlight and correct patterns of inequity [30-33]. Routine disaggregation of quality and outcome metrics by sex, age, and ethnicity allows organisations to monitor diagnostic intervals and investigation rates, identifying services where women are consistently under-investigated or diagnosed later than men [27-30,35].

Patient-facing interventions also have a role. Public health campaigns that highlight atypical symptom presentations in women, particularly for cardiovascular and neurological emergencies, can encourage timely presentation and self-advocacy [9-11]. Supporting primary care continuity, particularly for young women with complex or multisystem complaints, may reduce fragmentation and ensure that evolving symptom patterns are recognised early [4,18,34].

Finally, embedding gender equity within broader health policy, through research funding priorities, guideline development, and workforce training, can shift diagnostic culture over time. Addressing diagnostic gender bias in young women requires this multi-layered approach, integrating individual, organisational, and policy-level interventions to create a more equitable diagnostic landscape [1-4,19-21,30-37].

## Conclusions

Young women continue to experience diagnostic delays driven by gendered assumptions, atypical symptom framing, and male-centred diagnostic standards. These patterns have meaningful clinical consequences, including missed treatment windows and long-term health burden. Interventions such as sex-informed education, structured pathways, routine audit of diagnostic equity, and patient-centred communication can meaningfully reduce these disparities. Recognising diagnostic gender bias as a systemic issue requiring coordinated clinical and policy reform is essential for achieving earlier diagnosis and equitable care for young women.
